# Supplementary material for: Permeability of Metformin across an In Vitro Blood–Brain Barrier Model during Normoxia and Oxygen-Glucose Deprivation Conditions: Role of Organic Cation Transporters (Octs)
Source: Pharmaceutics. 2023 Apr 28;15(5):1357. doi: 10.3390/pharmaceutics15051357 (PMC10220878; doi:10.3390/pharmaceutics15051357)
Supplement: Supplementary file 1 [file pharmaceutics-15-01357-s001.zip › pharmaceutics-2316226-supplementary.pdf]

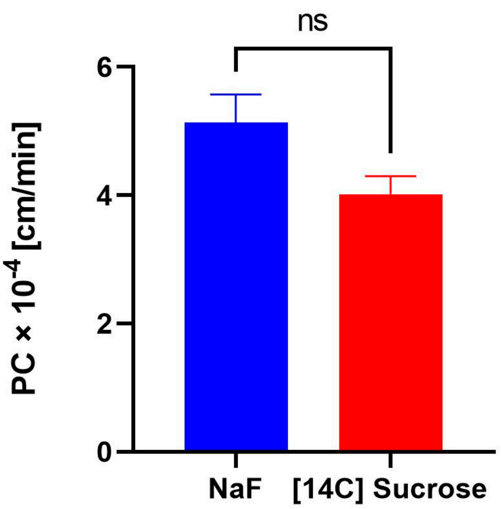

Figure S1. Permeability Coefficient (PC) of low molecular weight BBB markers, NaF (MW 376 Da), and [^{14}C] sucrose (MW 342 Da), across an *in vitro* co-culture setup of BBB. The PC of NaF compared to [^{14}C] sucrose was statistically non-significant. The experiments were performed under a similar setup of a co-culture model of brain endothelial cells and astrocytes. Unpaired student's t-test ($*p <$ 0.05); n= 3 biological replicates; mean ± SD.

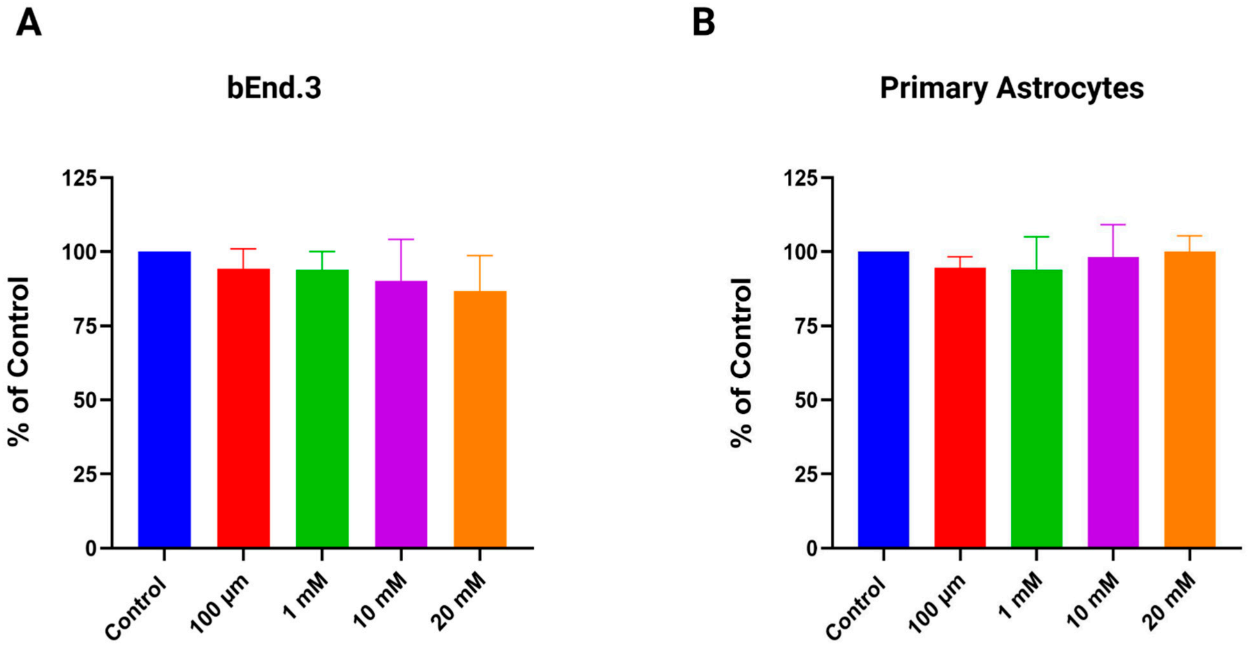

Figure S2. MTS cell viability assay in bEnd.3 cells **(A)** and mouse primary astrocytes **(B).** The values are expressed as a percentage of the control value. No statistically significant differences were found among the groups. One-way ANOVA, followed by Tukey's multiple comparisons tests; n= 3 biological replicates; mean ± SD.

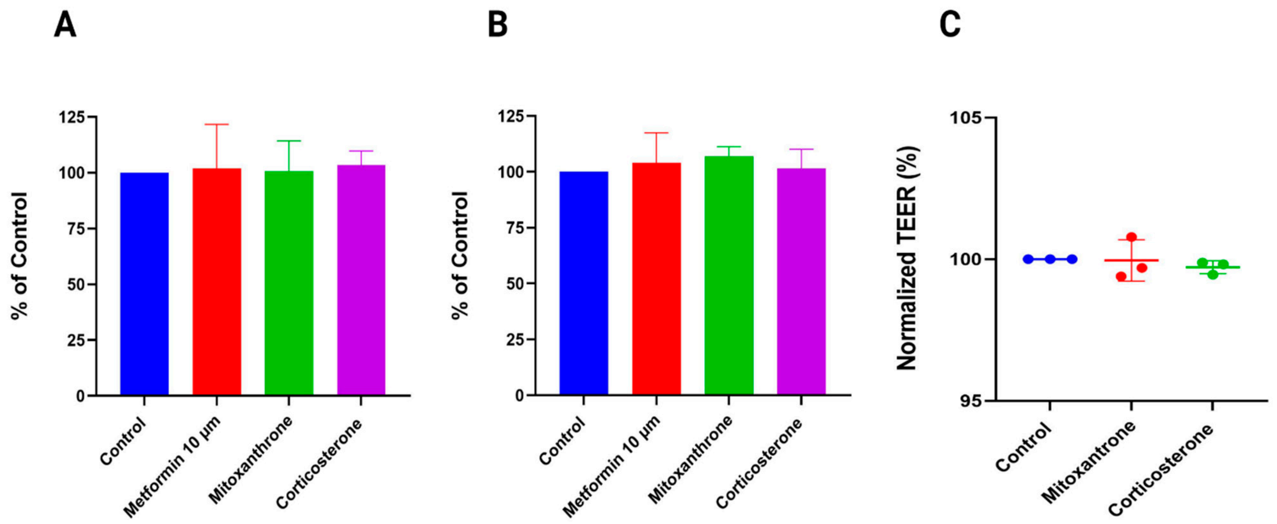

Figure S3. MTS cell viability assay in bEnd.3 cells **(A)** and mouse primary astrocytes **(B)**, and TEER measurement of co-cultured cells after incubation with inhibitors **(C)**. The values are expressed as a percentage of the control value. No statistically significant differences were found among the groups. One-way ANOVA, followed by Tukey's multiple comparisons tests; n= 2-3 biological replicates; mean ± SD.

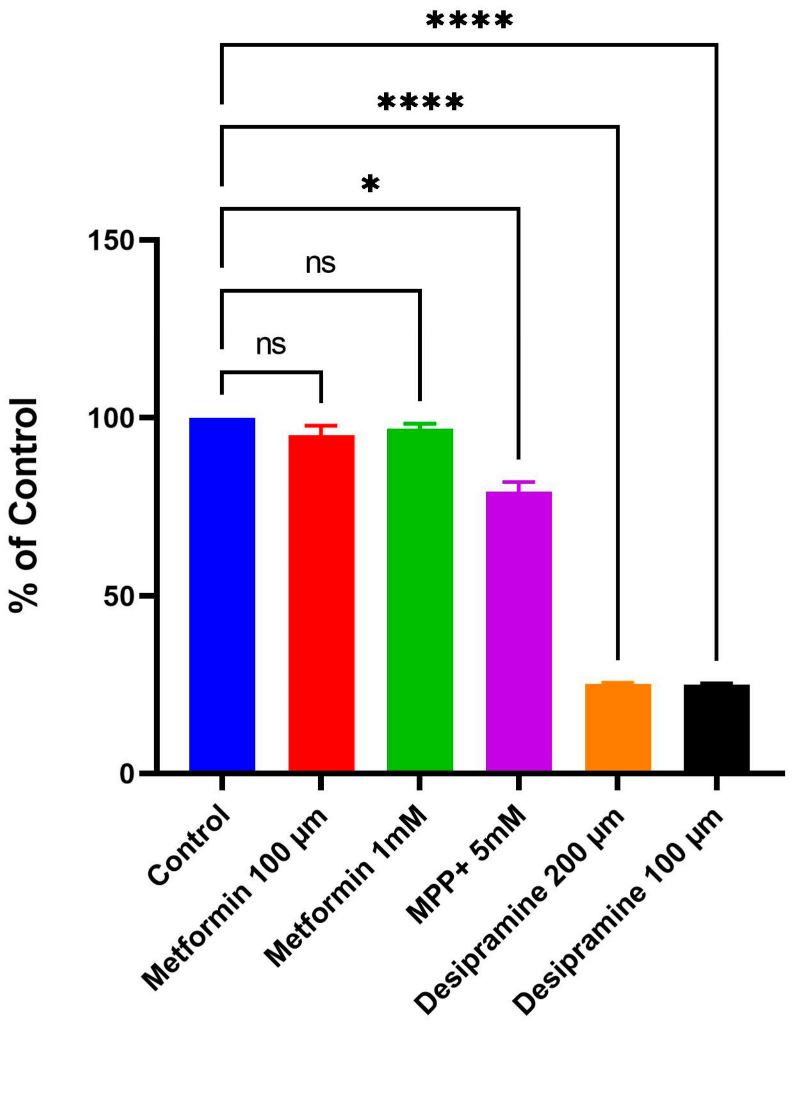

Figure S4. MTS cell viability assay in bEnd.3 cells using during 4 hours of incubation with substrates/ inhibitors. The values are expressed as a percentage of the control value. The cell viability was significantly affected with MPP+ at 5 mM concentration, and desipramine at 100 μm and 200 μm concentrations respectively. One-way ANOVA, followed by Tukey's multiple comparisons tests; n=3; mean ± SD.
